# Association of dietary diversity score and severity of pemphigus vulgaris: a cross-sectional study

**DOI:** 10.1186/s40795-025-01193-0

**Published:** 2025-11-11

**Authors:** Banafsheh Jafari Azad, Maryam Fallah, Zahra Esmaeily, Anahita Najafi, Kamran Balighi, Maryam Daneshpazhooh, Soraiya Ebrahimpour-Koujan

**Affiliations:** 1 Department of Cellular and Molecular Nutrition, School of Nutritional Sciences and Dietetics, Tehran University of Medical Sciences, Tehran, Iran; 2 Department of Clinical Nutrition, School of Nutritional Sciences and Dietetics, Tehran University of Medical Sciences, No: 44, Hojjat-dost Alley, Naderi St., Keshavarz Blvd, PO Box: 14155-6117, Tehran, Iran; 3 Department of Community Nutrition, School of Nutritional Sciences and Dietetics, Tehran University of Medical Sciences, Tehran, Iran; 4 School of Medicine, Tehran University of Medical Sciences, Tehran, Iran; 5 Autoimmune Bullous Diseases Research Center, Tehran University of Medical Sciences, Tehran, Iran; 6 Department of Dermatology, Razi Hospital, Tehran University of Medical Sciences, Tehran, Iran; 7 Cancer Research Center, Cancer Institute, Tehran University of Medical Sciences, Tehran, Iran

**Keywords:** Pemphigus vulgaris, Diet diversity, PDAI, DDS

## Abstract

**Background:**

Previous studies support the protective role of a balanced diet containing several foods and nutrients in controlling the autoimmune bullous disease. Dietary diversity score (DDS) is a measure of diet quality based on the number of different food groups consumed, which may influence immune function and inflammatory responses relevant to autoimmune diseases such as pemphigus vulgaris. The present study was designed to investigate the potential the association between DDS and the risk of high-severity Pemphigus Vulgaris (PV) disease in adult Iranian patients.

**Methods:**

A cross-sectional study was performed on 138 patients, aged 18–65 years, with confirmed diagnoses of PV in a referral university center for autoimmune bullous diseases. Dietary intakes were assessed using a 168-item semi-quantitative food frequency questionnaire. Anthropometric measures, biochemical markers, and sociodemographic characteristics were collected using standardized methods. DDS was defined according to the Diet Quality Index, revised. To assess PV severity, the pemphigus disease area index (PDAI) score was used. Logistic regression was used to evaluate the association between DDS and PDAI.

**Results:**

Mean (± standard deviation) DDS was 4.98 ± 1.21. After adjusting for potential confounders, patients in the third and second quartiles of DDS had lower odds for disease severity based on the PDAI score (OR: 0.14, 95% CI: 0.03–0.69 and OR: 0.18, 95% CI: 0.04–0.86; respectively) compared to the reference group. This inverse relationship was observed even after stratification by the oral lesion (OR _crude_: 0.27, 95% CI: 0.08–0.96). In addition, the probability of having a high PDAI score decreased with increasing adherence to the diversity score for fruit compared with those with the lowest adherence (OR _crude_: 0.40, 95% CI: 0.17–0.92).

**Conclusions:**

Our findings suggest that a diversified diet intake may be associated with a lower severity of disease in PV patients. However, Additional studies are required to replicate these findings.

**Supplementary Information:**

The online version contains supplementary material available at 10.1186/s40795-025-01193-0.

## Introduction

Pemphigus is characterized as a group of rare autoimmune chronic blistering epithelial disorders that affect both mucous membranes and the skin, mostly in individuals aged 45–65 years [1, 2]. Pemphigus vulgaris (PV) is one of the most common and severe subtypes of the blistering disorder [3]. The global incidence rates of PV range from 0.76 to 32.0 cases per million annually, and 10.0 in Iran [2, 4]. Several studies reported the association between PV and various chronic conditions, e.g., rheumatoid arthritis, diabetes mellitus type I, malignancies, neuropsychiatric diseases, and musculoskeletal autoimmune disorders [2]. Pemphigus Disease Area Index (PDAI) is among the recently developed scoring system that evaluates skin damage [5].

Genetic vulnerability and environmental factors are the factors suggested to play a role in developing PV [6]. Stress, certain drugs (thiol-based, phenol-based, etc.), and nutritional factors, including allium-based foods, were also contributing factors [7–10]. Protein, vitamins E, A, iron, zinc, and copper are suggested to play important roles in immune health, wound integrity and healing, and collagen formation [11–13]. Studies have shown that the growth of skin cells and wound healing are related to the quality of the diet [14]. The association of some foods with the onset and severity of PV disease and the involvement of the oral mucosa in patients can, per se, have a negative effect on the amount of food intake, variety, and quality of diet [15].

Although dietary components are important, the presence of many unknown compounds in foods and possible interactions between nutrients have made the study of the entire diet preferable to focusing on a single constituent [16]. In this regard, the Dietary Diversity Score (DDS) has recently become the preferred approach for representing the access of the population to a variety of food and also for evaluating the sufficiency of dietary intake [17].

Multiple studies suggested a link between skin health and nutritional factors. Low adherence to the Mediterranean diet is positively associated with psoriatic plaques [18, 19]. In a case-report study, a gluten-free diet ameliorated the latter signs and symptoms of PV patients, especially skin lesions [20]. In a recent study by Seifollahi et al., an insignificant inverse association was shown between higher DDS and lower cardiovascular disease risk in PV [21]. It seems that various diet-related factors contribute to the autoimmune bullous diseases ' onset, progression, exacerbation, and treatment. However, to the best of our knowledge, there is very limited information on the relation of DDS with the risk and severity of PV.

Because of the paucity of studies in this area, and considering Iran as one of the high-prevalence regions of PV [2] the present study aimed to classify PV patients based on the severity of the disease and to evaluate its relationship with dietary diversity among Iranian PV patients. The results obtained will be more important for patients residing in the Middle East, where limited information is available about diet and disease.

## Materials and methods

### Study design and participants

A hospital-based cross-sectional study involving diagnosed patients with PV was carried out in 2021. The adult PV patients are referred to the Pemphigus clinic of Razi Hospital, a referral center for skin disease in Iran. The diagnosis of PV has been confirmed based on a positive direct immunofluorescence test as the gold standard for PV by a pathologist at the Pemphigus clinic of Razi Hospital. One hundred and thirty-eight adult PV patients were selected without any gender restriction. Participants were patients with pathologic PV confirmation, 18–65 years of age. Exclusion criteria included being a migrant, pregnant, lactation, visiting a nutritionist during the past year, having malignancy problems, patients with under- or over-reported total energy intake (< 800 or 4200 > Kcal/day), and a history of inflammatory disease except for pemphigus vulgaris. The sample size in the present study was 138 cases, calculated using the specific formula for cross-sectional studies and considering types I and II error of 0.05 (N= [(Zα + Z_1−_)/0.5×ln(1 + r/1-r)]^2^ +3. Using 1-_β_ = 0.95 and α = 0.05, also a correlation coefficient of 0.3 was determined as the minimum acceptable correlation; *r* = 0.30). A written informed consent form with necessary declarations was obtained from all participants. The present study was approved by the ethics committee of the Tehran University of Medical Sciences (IR.TUMS.MEDICINE.REC.1400.1450) and was in accordance with the Helsinki Declaration.

### Assessment of dietary intake

Habitual dietary intake of PV patients during the last year of study enrollment was collected using a valid and reliable semi-quantitative food frequency questionnaire [22]. This questionnaire consists of 168 food items with standard serving sizes commonly consumed in Iran. Questionnaires were completed through face-to-face personal interviews with a trained dietitian via face-to-face interview. Patients were asked how often, on average, per week or per day, they had consumed a specific portion of each of 168 food items over the last 12 months. The amount of each portion size was converted to grams according to the household measures. Then the grams of consumption of each food item were converted to daily intake by dividing the weight of each food item consumed by a time factor (for example, once a month was divided by 30 and once a week was divided by 7). Average daily energy macronutrient and micronutrient consumption for participants was also calculated using an updated version of Nutritionist IV software, which was designed for the evaluation of Iranian foods (version 7.0; N-Squared Computing, Salem, OR, USA).

### Assessment of disease severity

The Pemphigus Disease Area Index (PDAI) score is an international pemphigus scoring system for defining moderate, significant, and extensive types of pemphigus based on the level of involvement in the skin or mucous membrane of different parts of the body. The validity and reliability of the PDAI score have been determined among adults [23, 24]. This score was used to evaluate the pemphigus severity by observational and physical examination by trained dermatology assistants. The PDAI score ranges is 0–263: 250 points evaluate the activity of the disease (10 points for the scalp, 120 for skin, 120 points for mucous membranes activity, and 13 points for post-inflammatory hyperpigmentation), representing disease damage [24]. After calculating the PDAI score for each patient based on the number and size of lesions in each anatomical area, the patients were divided into two groups: moderate (PDAI less than or equal to 15) and severe (PDAI greater than 15).

### Dietary diversity score (DDS) calculation

DDS is an index that reflects the number of different food groups consumed over a reference period, used as a proxy for diet quality and nutrient adequacy [25]. DDS was calculated by merging the methods of Kant and Haines et al. [17, 26, 27]. Foods were categorized into five main groups: dairy, grains, vegetables, fruits, and meat. Afterward, dairy products were divided into 3 subgroups (milk, cheese, yogurt), grains into 7 subgroups (refined bread, biscuits, macaroni, whole bread, cornflakes, rice, refined meal), vegetables into 7 subgroups (vegetables, potatoes, tomatoes, starchy vegetables, legumes, yellow vegetables, green vegetables), fruits into 2 subgroups (fruit and fruit juice, berries and citrus), and meats into 4 subgroups (red meats, chicken, poultry, eggs, fish). Accordingly, 23 subgroups were formed. To be counted as a “consumer”, at least one-half serving of every subgroup’s food list should be consumed based on the Food Guide Pyramid quantity criteria by a subject. No need to eat it all at once. The total DDS ranged from 0–10, and each one of the groups gets a maximum of 2 points, and the sum of the groups’ points constitutes the total score of dietary diversity. The score of each group was determined by the division of the number of subgroups consumed by the total number of subgroups in each main group, and finally, it was multiplied by 2. For instance, in the meat group, if someone consumed just half of a serving of red meat, the score was calculated as (1 ÷ 4) × 2 = 0.5; thereby, the diversity score of the meat group is 0.5 [27]. DDS is categorized into quartiles to distinguish different levels of diversity of the diet.

### Assessment of other variables

Participants were asked about the general characteristics and lifestyle variables including age (continuous), gender (male/female), marital status (single/married), education (under university/university graduated), duration of disease (continuous), being in a high-risk job (exposing to chemicals/none exposing to chemicals), smoking (smoker/non-smoker). Also, information about medical history such as rituximab cumulative dose (continuous (, having a history of any chronic diseases including diabetes, hypertension, cardiovascular disease, hyperlipidemia, cancer, hypothyroidism, non-alcoholic fatty liver disease, polycystic ovary syndrome, viral infections and family history of the mentioned disease were obtained using a general demographic questionnaire.

Anthropometric parameters were taken according to the World Health Organization recommendations. Weight (nearest 100 g) was measured with minimal clothing and without shoes using a digital scale (SECA, Hamburg, Germany), and height (nearest 0.1 cm) was measured using a non-elastic tape meter while standing next to the wall, the shoulders were in a normal state, and barefoot. Waist circumference was measured using a non-stretching tape measure at the midpoint between the last rib and the sternum. BMI was computed as the patients’ weights divided by height squared (kg m^–2^). All anthropometric indices were measured by a trained GP. Physical activity was assessed using a validated international physical activity questionnaire (IPAQ) and expressed as metabolic equivalents ^×^ hours per day, and then categorized into mild, moderate, and intensive [28].

### Statistical analysis

The statistical analysis was conducted using SPSS software (SPSS Inc., Chicago, IL - version 22.0). The level of statistical significance was set as P values less than 0.05. Categorical variables were expressed as numbers and percentages. Continuous variables were presented as mean ± SD/SE. All continuous data were tested for normality by the Kolmogorov-Smirnov test. The patients were categorized based on DDS and PDAI scores. Comparison of general characteristics and dietary intakes between the two categories of PDAI score was performed using an Independent Sample *t-test* and Chi-square or Fisher’s exact tests for continuous and ordinal qualitative variables, respectively. Comparison of these characteristics across the quartiles of DDS was done using the One-way analysis of variance (ANOVA) test and Chi-square or Fisher’s exact tests. A binary logistic regression test was used to investigate the association between total DDS and its components with the risk of severity of the disease based on the PDAI score components in crude and adjusted models. PDAI by coding (PDAI ≤ 15 = 0, PDAI > 15 = 1 as dummy variable) is considered as a dependent (Y) variable in the regression model. In the multivariable model, the potential confounding variables including age, gender, energy intake, waist-circumference, high-risk job, education, marital status, residence, physical activity, smoking, duration of disease, past medical history, supplement use, fasting blood sugar, cholesterol, rituximab, azathioprine, methotrexate were adjusted in six separate models. In all multivariate models, Q1 of the DDS was considered as the reference. Logistic regression was used to investigate the association between total DDS and severity of cutaneous and mucous membrane involvement in crude and adjusted models. Also, the linear regression analysis was used to predict the severity of PV disease following a one-unit increase in DDS.

## Results

**Fig. 1:**
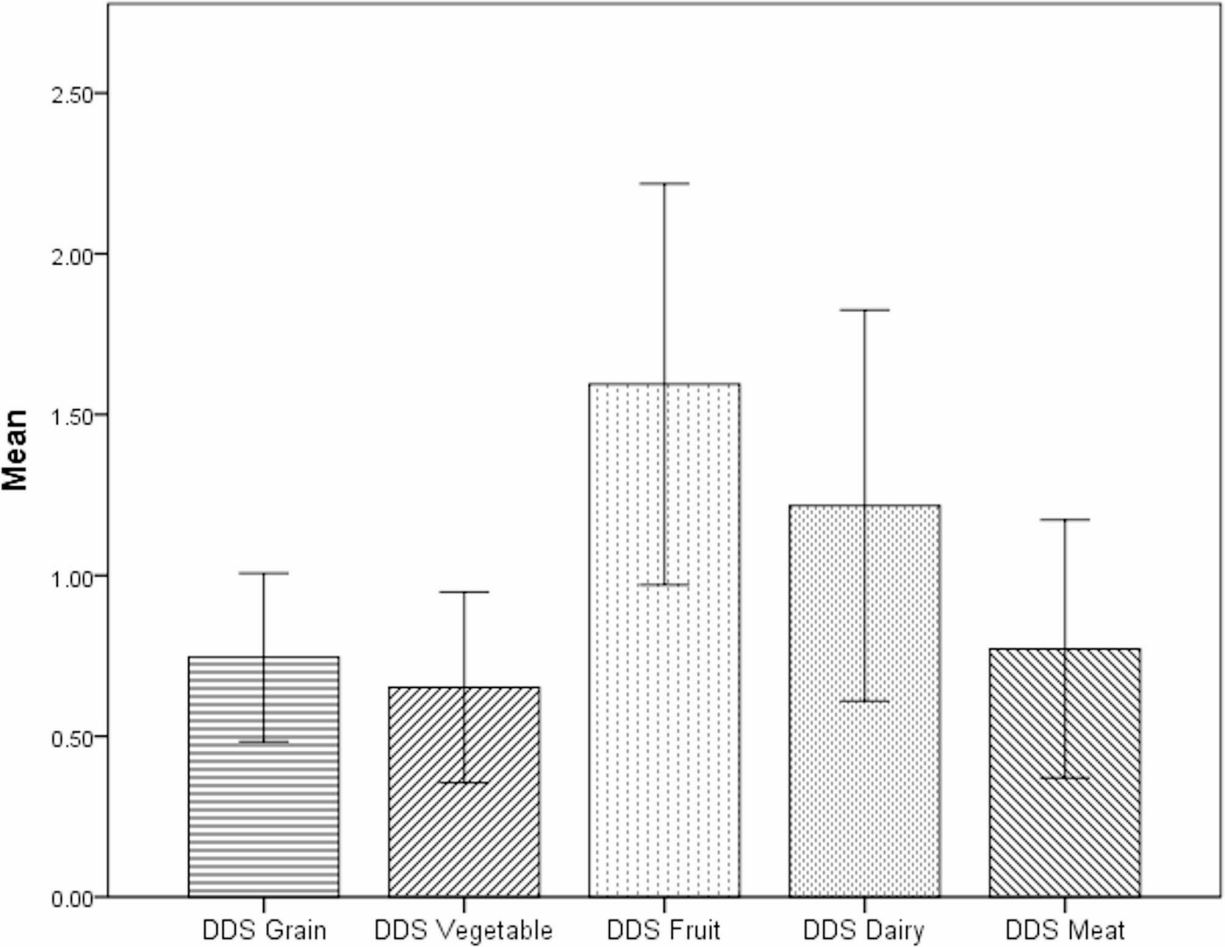
Distribution of the dietary diversity score

**Fig. 2:**
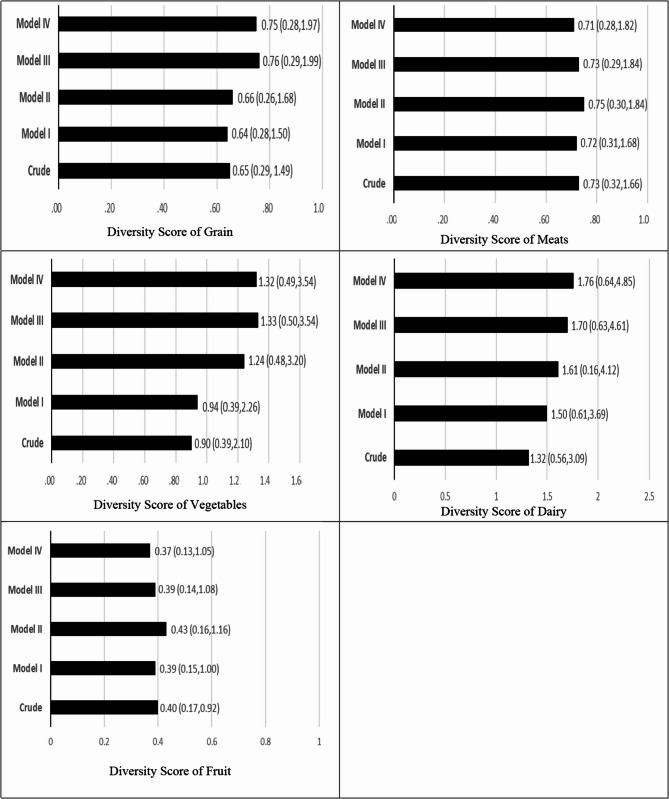
Odds ratio (95% confidence interval) of pemphigus disease area index (PDAI) score, by ranks of the diversity score for grains, vegetables, dairy, fruits, and meats. OR (CI95%) for highest versus lowest. According to the median dietary diversity the participants were dichotomized into “high” and “low” adherence (≤, >of median): grains (≤ 0.86, 0.87>), vegetables (≤ 0.57, 0.58>), meats (≤ 0.50, 0.51>), dairy (≤ 1.33, 1.34>), fruit (≤ 2.00, 2.1>). PDAI scores divided into two groups mild (PDAI ≤ 15) and severe (PDAI > 15). Model I: adjusted for kcal, age, and gender. Model II: adjusted for all variables in model I and high-risk job, education, marital status, smoking, duration of disease, and past medical history. Model III: adjusted for all variables in model II and total cholesterol and rituximab. Model IV: adjusted for all variables in model III and body mass index

A total of 138 PV patients were included in the study. Food items were divided into 5 main groups: grains, vegetables, fruits, meat, and dairy products. The distribution of the dietary diversity score is mentioned in Fig. 1. The mean ± standard deviation of the total DDS was 4.98 ± 1.21. The maximum and minimum scores for diversity were related to fruit (1.59 ± 0.62) and vegetable (0.65 ± 0.29) groups, respectively. Total DDS is divided into 4 quartiles: less than 3.98, 3.99–5.00, 5.01–5.88, and more than 5.89. The odds ratio (95% confidence interval) of the PDAI score by ranks of the diversity score for grains, vegetables, dairy, fruits, and meats is mentioned in Fig. 2.

This study investigated the associations between DDS and PDAI scores in patients with PV. The results are organized first to describe the characteristics of the study population (Table 1), followed by dietary intakes (Table 2), associations between DDS and PDAI scores (Table 3). Key findings highlight the inverse association between DDS and disease severity, particularly in the second and third quartiles of DDS.

**Table 1:** Characteristics of the study population by two categories of DDS and PDAI scores

Variables	PDAI scores			DDS Quartile Categories				
	PDAI < = 15	PDAI > 15	*P* a	Q1	Q2	Q3	Q4	*P* a
Number of Patients	*n* = 108	*n* = 30		35	34	33	36	
Demographic								
Age (year) c	48.24(12.44)	48.33(14.32)	0.972	49.14(13.06)	47.32(13.74)	52.57(12.74)	44.33(10.75)	0.057
Gender Male/Female b	41/67(18%,62%)	15/15(50%,50%)	0.235	16/19(45.7%,54.3%)	14/20(41.2%,58.8%)	15/18(45.5%,54.5%)	11/15(30.6%,69.4%)	0.529
Weight (kg) c	76.31(14.50)	76.8(12.22)	0.869	76.57(15.12)	75.80(12.76)	74.54(14.98)	78.58(13.33)	0.682
WC (cm) c	86.94(12.23)	85.56(12.46)	0.588	86.65(11.94)	83.64(9.37)	86.57(12.62)	89.52(14.24)	0.259
BMI (kg/m^2^) c	27.98(4.73)	27.15(4.44)	0.390	27.90(4.69)	26.88(3.73)	27.40(5.32)	28.92(4.75)	0.303
Smoking yes/no b	12/96(11.1%,88.9%)	6/24(20%,80%)	0.224	5/30(14.3%,85.7%)	5/29(14.7%,85.3%)	2/31(6.1%,93.9%)	6/30(16.7%)	0.587
PAL b								
Milde	45(41.7%)	13(43.3%)	0.855	15(42.9%)	13(38.2%)	12(36.4%)	18(50%)	0.484
Moderate	48(44.4%)	14(46.7%)		18(51.4%)	14(41.2%)	15(45.5%)	15(41.7%)	
Intensive	15(13.9%)	3(10%)		2(5.7%)	7(20.6%)	6(18.2%)	3(8.3%)	
University-Education yes/no b	32/76(29.6%,70.4%)	7/23(23.3%,76.7%)	0.498	8/27(22.9%,77.1%)	7/27(20.6%,79.4%)	13/20(39.4%,60.6%)	11/25(30.6%,69.4%)	0.308
Marital Status.Single/Marid b	15/93(13.9%,86.1%)	6/24(20%,80%)	0.400	2/33(5.7%,94.3%)	9/25(26.5%,73.5%)	6/27(18.2%,81.8%)	4/32(11.1%,88.9%)	0.09
High-Risk Job yes/no b	4/104(3.7%,96.3%)	4/26(13.3%,86.7%)	0.068	1/34(2.9%,97.1)	5/29(14.7%,85.3%)	2/31(6.1%,93.9%)	0/36(0%,100%)	0.038
Residence Urban/Rural b	101/7(93.5%,6.5%)	29/1(96.7%,3.3%)	1.000	34/1(97.1%,2.9%)	33/1(97.1%,2.9%)	31/2(93.9%,6.1%)	32/4(88.9%,11.1%)	0.498
Medical History								
Disease Duration(day) c	5.37(6.04)	2.55(3.71)	0.002	4.80(6.32)	3.98(4.98)	5.40(6.35)	4.85(5.30)	0.794
PMH yes/no b	62/46(57.4%,42.6%)	17/13(56.7%,43.3%)	0.942	22/13(62.9%,37.1%)	19/15(55.9%,44.1%)	22/11(66.7%,33.3%)	16/20(44.4%,55.6%)	0.253
Supplemet Use. yes/no b	17/91(15.7%,84.3%)	3/27(10%,90%)	0.565	2/33(5.7%,94.3%)	7/27(20.6%,79.4%)	4/29(12.1%,87.9%)	7/29(19.4%,80.6%)	0.233
Corticosteroid Medication (Current dose) c	9.79(9.12)	28(15.97)	< 0.0001	16.12(14.66)	13.93(13.74)	11.68(10.93)	13.15(13.49)	0.576
RTX Medication (Cumulative dose) c	2.73(2.55)	1.53(1.70)	0.016	2.88(2.93)	2.58(2.79)	2.13(1.87)	2.27(2.00)	0.589
MTX Medication yes/no b	6/102(5.6%0.94.4%)	0/30(0%,100%)	0.339	0/35(0%,100%)	3/31(8.8%,91.2%)	2/31(6.1%,93.9%)	1/35(2.8%,97.2%)	0.229
Azathioprine yes/no b	1/107(0.9%,99.1%)	2/28(6.7%,93.3%)	0.119	0/35(0%,100%)	1/33(2.9%,97.1%)	1/32(3%,97%)	1/35(2.8%,97.2%)	0.803
Systolic BP (mmHg) c	123.77(10.23)	125.66(10.96)	0.380	125.51(11.79)	120.88(10.76)	124.39(8.08)	125.83(10.15)	0.177
Diastolic BP (mmHg) c	76.64(6.56)	77.16(7.51)	0.711	77.65(9.15)	76.02(5.47)	75.96(6.03)	77.30(5.77)	0.641
Lab Test								
FBS (mg/dl) c	95.86(19.26)	103.36(37.77)	0.301	92.4(2.77)	98.26(3.31)	101.09(6.44)	98.58(3.87)	0.517
LDL (mg/dl) c	103.56(30.09)	105.83(32.43)	0.720	108.02(4.39)	112.79(5.32)	95.42(5.13)	99.86(5.56)	0.080
HDL (mg/dl) c	54.04(11.62)	52.46(10.98)	0.506	53.8(2.06)	55.38(2.14)	53.69(1.68)	52.02(1.92)	0.686
TG (mg/dl) c	131.15(61.02)	157.03(72.98)	0.054	128.55(11.19)	150.76(12.65)	132.84(10.30)	134.38(9.80)	0.510
TC (mg/dl) c	183.68(35.96)	187.76(41.17)	0.595	185.62(5.61)	199.17(7.21)	172.81(5.60)	180.52(6.08)	0.027
Anti-DSG1 Positive/Negative b	24/84(22.2%,77.8%)	11/19(36.7%,63.3)	0.108	10/25(28.6%,71.4%)	6/28(17.6%,82.4%)	7/26(21.2%,78.8%)	12.24(33.3%,66.7%)	0.225
Anti-DSG3 Positive/Negative b	51/41(55.4%,44.6%)	12/3(80%,20%)	0.073	16/9(64%36%)	14/12(53.8%,46.2%)	15/9(62.5%,37.5%)	18/14(56.3%,43.8%)	0.599
PDAI Score	-	-	-	12.51(13.54)	7(7.26)	4.78(6.00)	8.25(12.19)	0.020

a chi-square test for ordinal qualitative variables, Independent Samlpe T test and ANOVA test with Tukey’s test for continuous variables, b N (%), c Mean ± SD, d Mean ± SE

*WC* Waist circumference, *BMI* Body mass index, *PAL* Physical activity level, *PMH* Past medical History, *PDAI* Pemphigus disease area index, *RTX* Rituximab, *MTX* Methotrexate, *FBS* Fasting blood sugar, *LDL* Low density lipoprotein, *HDL* High density lipoprotein, *TG* Triacylglycerol, *TC* Total cholesterol, *PMH* Diabete hypertansion Cardiovascular disease hyper-lipidemia cancer hypothyroidism non-fatty liver disease, *RTX Medication* Ritoximb cumulative dose, *Anti-DSG1* Anti-desmoglein1 antibodies, *Anti-DSG3* Anti-desmoglein3 antibodies

Supplemet of vitamins and mineralsCorticosteroid Medication: Dose of the current use of prednisolone

**Table 2:** Daily dietary intake of the study population between categories of DDS and PDAI scores

	PDAI score		*P* ^a^	Quartile of total dietary diversity score				*P* ^b^
	(score < = 15)	(score > 15)		Q1	Q2	Q3	Q4	
Kcal*(kcal/d)	2283.76(61.81)	2275.71(122.76)	0.952	1817.05(88.29)	2258.10(98.47)	2432.22(97.87)	2618.93(107.54)	< 0.0001
Macronutrients								
Protein(g/day)	90.01(28.52)	91.90(25.89)	0.743	68.14(19.90)	83.42(17.36)	98.06(22.08)	111.68(31.19)	< 0.0001
CHO(g/day)	327.09(97.31)	323.65(115.21)	0.870	263.01(14.83)	323.94(13.47)	344.92(17.73)	373.16(17.14)	< 0.0001
Fat(g/day)	74.65(28.10)	73.31(27.37)	0.817	58.12(3.63)	76.48(5.67)	79.59(3.94)	83.36(4.45)	< 0.0001
Fat-subgroup								
Cholestreol(mg/day)	267.00(124.01)	334.59(186.15)	0.020	207.15(13.89)	257.20(16.96)	296.82(17.04)	363.44(33.76)	< 0.0001
SFA(g/day)	22.21(9.58)	22.59(9.97)	0.852	15.82(1.10)	23.65(1.84)	24.201.42)	25.56(1.61)	< 0.0001
MUFA(g/day)	21.78(9.41)	21.65(8.480	0.942	16.97(1.37)	22.03(1.68)	23.15(1.35)	24.85(1.55)	0.002
PUFA(g/day)	15.48(7.64)	14.16(5.98)	0.384	13.70(6.63)	15.98(9.08)	15.98(7.09)	15.19(6.28)	0.531
Oleic(g/day)	19.19(9.82)	17.59(7.72)	0.411	14.85(8.95)	18.85(9.17)	18.83(6.91)	22.74(10.65)	0.005
Linoleic(g/day)	12.84(7.34)	11.96(5.80)	0.547	12.00(6.61)	13.54(8.55)	12.87(7.12)	12.22(5.85)	0.802
Linolenic(g/day)	0.10(0.11)	0.14(0.12)	0.147	0.07(0.04)	0.15(0.14)	0.11(0.09)	0.12(0.14)	0.024
EPA(g/day)	0.03(0.04)	0.03(0.04)	0.974	0.02(0.03)	0.02(0.03)	0.03(0.03)	0.04(0.06)	0.319
DHA(g/day)	0.07(0.10)	0.07(0.09)	0.974	0.05(0.06)	0.06(0.08)	0.07(0.07)	0.09(0.14)	0.319
Minerals								
Iron(mg/day)	20.50(9.38)	21.25(14.18)	0.073	14.56(4.61)	20.31(8.29)	20.80(8.94)	26.80(14.19)	< 0.0001
Calcium(mg/day)	1060.47(348.82)	1074.73(375.87)	0.846	823.24(37.32)	1005.22(48.98)	1123.63(61.49)	1297.27(60.14)	< 0.0001
Magnesium(mg/day)	289.76(85.81)	267.24(72.85)	0.192	216.29(10.83)	272.98(11.02)	299.97(12.67)	348.92(12.08)	< 0.0001
Phosphoros	1321.76(422.64)	1347.22(429.97)	0.772	1007.76(46.85)	1296.40(73.89)	1375.21(58.66)	1623.20(64.76)	< 0.0001
Zinc(mg/day)	9.52(3.21)	9.49(3.29)	0.957	7.00(2.01)	9.07(2.61)	9.91(2.06)	12.03(3.62)	< 0.0001
Coper(mg/day)	1.39(0.64)	1.24(0.55)	0.229	1.00(0.44)	1.41(0.64)	1.40(0.55)	1.62(0.66)	< 0.0001
Selenium(mg/day)	0.03(0.03)	0.05(0.04)	0.108	0.03(0.03)	0.04(0.03)	0.04(0.03)	0.04(0.03)	0.276
Sodium*	4814.47(299.19)	4330.99(364.24)	0.311	4418.36(445.44)	4898.3(416.66)	4641.76(422.80)	4875.83(285.78)	0.804
Potasium* (mg/d)	3727.81(110.39)	3339.45(179.41)	0.094	2683.66(137.35)	3496.34(142.41)	3896.51(179.26)	4483.30(160.99)	< 0.0001
Vitamin-Fat soluble								
VitA*(µg/d)	1604.76(97.68)	1420.57(184.56)	0.380	1069.32(164.85)	1330.44(126.14)	1525.13(129/60)	2303.91(183.56)	< 0.0001
Betacaroten	993.48(908.74)	827.03(794.36)	0.364	689.76(161.81)	720.15(87.54)	895.05(113.95)	1498.42(175.59)	< 0.0001
VitE(mg/day)	4.27(1.78)	4.22(1.38)	0.889	3.33(1.55)	3.95(1.31)	4.6(2.14)	5.08(1.15)	< 0.0001
Alpha-tocopherol	10.37(8.08)	8.84(7.30)	0.351	7.93(7.13)	11.98(10.52)	10.02(6.75)	10.26(6.47)	0.210
VitD(mg/day)	1.94(1.44)	2.38(1.74)	0.156	1.44(1.13)	1.80(1.46)	2.21(1.59)	2.68(1.61)	0.004
VitK	204.93(107.36)	205.89(111.20)	0.966	174.77(25.50)	179.35(10.35)	196.11(13.25)	267.31(15.82)	< 0.0001
Vitamin-Water soluble								
VitB1(mg/day)	1.76(0.54)	1.76(0.59)	0.965	1.45(0.45)	1.74(0.43)	1.8(0.57)	2.02(0.59)	< 0.0001
VitB2(mg/day)	1.73(0.57)	1.87(0.59)	0.242	1.36(0.37)	1.64(0.53)	1.86(0.48)	2.19(0.56)	< 0.0001
VitB3(mg/day)	21.31(7.08)	21.42(6.25)	0.936	17.19(5.07)	20.41(5.42)	22.93(7.08)	24.78(7.39)	< 0.0001
VitB5(mg/day)	6.06(2.00)	5.88(1.90)	0.665	4.46(1.36)	6.00(2.01)	6.35(1.58)	7.25(1.82)	< 0.0001
VitB6(mg/day)	1.58(0.65)	1.37(0.55)	0.114	1.05(0.40)	1.56(0.69)	1.61(0.57)	1.90(0.55)	< 0.0001
VitB7(mg/day)	26.50(9.35)	27.99(9.96)	0.448	20.56(6.35)	25.66(7.65)	27.64(9.40)	33.28(9.57)	< 0.0001
VitB9 (µg/day)	345.17(113.68)	314.96(103.03)	0.191	263.02(16.24)	314.38(13.48)	348.40(18.41)	425.98(16.38)	< 0.0001
VitB12(µg/day)	4.83(3.80)	5.57(3.48)	0.338	3.45(0.24)	4.56(0.65)	5.08(0.45)	6.81(0.86)	0.001
VitC(mg/day)	178.88(102.39)	114.80(61.33)	0.001	101.21(8.69)	163.99(11.87)	188.47(24.08)	206.27(13.95)	< 0.0001
Fiber-subgroup								
Fiber(g/day)	19.14(6.88)	15.61(5.79)	0.011	12.90(4.99)	17.85(4.54)	19.87(6.70)	22.79(6.61)	< 0.0001
Soliuble_fiber(g/day)	0.88(0.44)	0.68(0.40)	0.032	0.51(0.28)	0.83(0.34)	0.92(0.47)	1.07(0.45)	< 0.0001
Insol_fiber(g/day)	4.70(2.24)	3.48(1.90)	0.007	2.95(1.64)	4.28(1.78)	5.07(2.25)	5.45(2.33)	< 0.0001

^a^ Independent Sample T test, ^b^ ANOVA test with Tukey’s test

Mean ± SD

* Mean ± SE

**Table 3.** Odds ratio (OR) and 95 % confidence interval (95 % CI) of pemphigus disease area index (PDAI) score, according to quartile of total dietary diversity score (DDS)

	**Total dietary diversity score**				
	**Q1**	**Q2**	**Q3**	**Q4**	***P ***trend
Crude	1.00 (Ref)	0.41(0.13,1.26)	0.26(0.07,0.92)	0.54(0.19,1.56)	0.191
Model I	1.00 (Ref)	0.37(0.11, 1.23)	0.21(0.05,0.85)	0.49(0.14,1.74)	0.214
Model II	1.00 (Ref)	0.18(0.04,0.75)	0.18(0.04,0.80)	0.56(0.14,2.21)	0.406
Model III	1.00 (Ref)	0.14(0.03,0.69)	0.18(0.03,0.85)	0.56(0.13,2.33)	0.477
Model IV	1.00 (Ref)	0.14(0.03,0.69)	0.18(0.04,0.86)	0.56(0.13,2.33)	0.482
Disease subtype Having oral lesion					
Crude	1.00 (Ref)	0.41(0.13,1.26)	0.26(0.07,0.92)	0.54(0.19,1.56)	0.191
Model I	1.00 (Ref)	0.37(0.11, 1.23)	0.21(0.05,0.85)	0.49(0.14,1.74)	0.214
Model II	1.00 (Ref)	0.18(0.04,0.75)	0.18(0.04,0.80)	0.56(0.14,2.21)	0.406
Model III	1.00 (Ref)	0.14(0.03,0.69)	0.18(0.03,0.85)	0.56(0.13,2.33)	0.477
Model IV	1.00 (Ref)	0.14(0.03,0.69)	0.18(0.04,0.86)	0.56(0.13,2.33)	0.482

Logistic regression models were used to estimate ORs with 95% CIs

The Low adherence to dietary diversity is the reference category in the logistic regression model

P-trend is resulted from logistic regression test

Model I: adjusted for kcal, age, gender

Model II: adjusted for all variables in model I and high-risk job, education, marital status, smoking, duration of disease, past medical history

Model III: adjusted for all variables in model II and total cholesterol and rituximab

Model IV: adjusted for all variables in model III and body mass index

Table 1 presents the demographic and clinical characteristics of pemphigus vulgaris (PV) patients stratified by PDAI scores and DDS quartiles. Patients with higher PDAI scores (> 15) had a significantly shorter disease duration (2.55 ± 3.71 vs. 5.37 ± 6.04 days, *P* = 0.002) and higher corticosteroid doses (28.00 ± 15.97 vs. 9.79 ± 9.12 mg/day, *P* < 0.001). Additionally, a lower prevalence of high-risk occupations was observed in the highest DDS quartile (0% in Q4 vs. 2.9% in Q1, *P* = 0.038), which may reflect lifestyle factors supporting greater dietary diversity, potentially linked to the protective effects observed in Q2 and Q3 (Table 3). No significant differences were found in other variables, such as BMI (27.15 ± 4.44 vs. 27.98 ± 4.73 kg/m², *P* = 0.390), blood pressure, or lipid profiles across PDAI scores or DDS quartiles. The general characteristics of the PV patients based on PDAI scores and across DDS scores are presented in Table 1. Across categories of the PDAI score, patients with high PDAI scores had a shorter duration of disease and higher doses of prednisolone. Having high-risk occupations was significantly lower among patients in the highest ranking of dietary diversity compared to those in the lowest (*P* = 0.038). There was no regular trend across the quartiles of DDS and levels of total cholesterol and PDAI score. However, it can be seen that these indices have lower levels in the third quarter. There were no other significant differences in other variables across categories of PDAI score and DDS quartiles. Having established the population characteristics, we next examined daily dietary intake to explore its relationship with PDAI scores and DDS quartiles. Table 2 summarizes the daily dietary intake of the study population, stratified by PDAI scores and DDS quartiles. Among patients with higher PDAI scores (> 15), significantly lower intakes of total fiber (15.61 ± 5.79 vs. 19.14 ± 6.88 g/day, *P* = 0.011), soluble fiber (0.68 ± 0.40 vs. 0.88 ± 0.44 g/day, *P* = 0.032), insoluble fiber (3.48 ± 1.90 vs. 4.70 ± 2.24 g/day, *P* = 0.007), and vitamin C (114.80 ± 61.33 vs. 178.88 ± 102.39 mg/day, *P* = 0.001) were observed, alongside a higher intake of dietary cholesterol (334.59 ± 186.15 vs. 267.00 ± 124.01 mg/day, *P* = 0.020). These findings suggest that reduced consumption of fiber-rich foods and vitamin C, coupled with elevated cholesterol intake, may be associated with greater disease severity in patients with PV. Across DDS quartiles, patients in the highest quartile (Q4) exhibited significantly higher intakes of energy (2618.93 ± 107.54 kcal/day, *P* < 0.001), macronutrients (protein: 111.68 ± 31.19 g/day, *P* < 0.001; carbohydrates: 373.16 ± 17.14 g/day, *P* < 0.001; total fat: 83.36 ± 4.45 g/day, *P* < 0.001), and most micronutrients, including iron (26.80 ± 14.19 mg/day, *P* < 0.001), calcium (1297.27 ± 60.14 mg/day, *P* < 0.001), magnesium (348.92 ± 12.08 mg/day, *P* < 0.001), and vitamin A (2303.91 ± 183.56 µg/day, *P* < 0.001), compared to those in the lowest quartile (Q1). However, intakes of polyunsaturated fatty acids (PUFA: 15.19 ± 6.28 g/day in Q4, *P* = 0.531), selenium (0.04 ± 0.03 mg/day in Q4, *P* = 0.276), and sodium (4875.83 ± 285.78 mg/day in Q4, *P* = 0.804) did not differ significantly across DDS quartiles, indicating that these nutrients may not be influenced by dietary diversity in this population. Notably, patients in the third quartile (Q3) showed a balanced increase in nutrient intake, which may contribute to the inverse association with PDAI scores observed in this quartile (Table 3).

We next investigated the association between DDS and disease severity using logistic regression models. Table 3 presents the multivariable-adjusted odds ratios (ORs) and 95% confidence intervals (CIs) for the association between DDS quartiles and PDAI scores. A key finding was the significant inverse association between DDS and disease severity in the second and third quartiles. In the crude model, patients in Q3 had 74% lower odds of high PDAI scores (OR = 0.26; 95% CI: 0.07–0.92; *P* = 0.031), and this association strengthened after full adjustment in Model IV (OR = 0.18; 95% CI: 0.04–0.86; *P* = 0.031). Similarly, in Q2, patients were 86% less likely to have high disease severity after adjusting for demographic factors, medical history, total cholesterol, rituximab, and BMI (OR = 0.14; 95% CI: 0.03–0.69; *P* = 0.016). Linear regression analysis further supported a dose-response relationship, with a one-unit increase in DDS associated with a 1.60-unit decrease in PDAI score (β = −1.60; *P* = 0.030).

In stratified analyses for oral lesions, similar inverse associations were observed in Q2 (OR = 0.14; 95% CI: 0.03–0.69; *P* = 0.016) and Q3 (OR = 0.18; 95% CI: 0.04–0.86; *P* = 0.031) in Model IV, although the association in Q3 weakened after adjustment for total cholesterol and rituximab (OR = 0.21; 95% CI: 0.04–1.00; *P* = 0.050).

These findings underscore the potential role of dietary diversity, particularly in moderate levels (Q2 and Q3), in reducing PV severity, highlighting the need for further research into dietary interventions for PV management.

## Discussion

In the present cross-sectional report, we did not reach any significant association between DDS and the severity of PV. However, an inverse association was observed between adherence to DDS and severity of PV in the third quartile in both the general population and in stratified analyses by oral lesion. A balanced and diversified diet in patients leads us to think that it could be a protective factor against the severity of the disease, but more studies are needed.

DDS reflects a complete view of diet quality and nutrient adequacy and is a useful indicator for determining the relationship between diet and diseases [29]. Previous studies have suggested that diets with high DDS have protective effects against chronic diseases [30–32]. Some studies indicated that monotonous diets with a lower DDS were associated with nutrient deficiencies and reflected poor diet quality [33, 34]. In the present study, we observed that the consumption of healthier foods and the intake of micronutrients and macronutrients were higher in the top categories of DDS.

The Mean ± SD of dietary diversity score (4.98 ± 1.21) was obtained. The highest and lowest DDS were related to the fruits and vegetables group (1.59 ± 0.63) and (0.65 ± 0.30), respectively. In the present study, the mean of total DDS showed less food variety compared with other studies in healthy Iranian adults [27, 35, 36]. However, the values were almost similar to those of postmenopausal Korean women [37]. Further looking into Iranian diets, there is a wide variety of vegetables and fruits in Iran. Previous studies on the status of dietary diversity among Iranians also showed that the highest score belongs to the group of fruits, and then vegetables [38, 39]. In this study, lower DDS scores may be due to mucous membrane involvement, which could affect the choice, variety of food, and the amount of intake. On the other hand, one epidemiologic study reported that Iranian households consumed more vegetables in summer and spring [40]. Since our data collection was in the winter, seasonal variation may have influenced the type, quality, and accessibility of vegetables and could be considered as another reason for the DDS-vegetables group having a low score in our study. In addition, in accord with ours, the results of a systematic review indicated an inverse association between vegetable consumption and the winter season, whereas the intake of fruits did not exhibit any notable seasonal fluctuations [41].

Although the exact mechanism underlying diet and PV severity is not well defined, there are some explanations. PV is a mucocutaneous autoimmune bullous disease, in which there is a loss of epidermal and mucosal cell-cell junctions [42]. Predominantly, IgG autoantibodies against epidermal adhesion proteins induce loss of cell integrity through activating inflammatory pathways such as p38 MAPK (Mitogen-activated protein kinase) signaling, resulting in acantholysis as well as activation of NF-kB (Nuclear factor-κB) and CREB proteins (cAMP-response element binding) [43]. Therefore, it seems that inflammation plays a crucial role in the development of this disease.

In the present study, we observed significant reverse associations between the DDS score and PDAI score in the third quartile. However, no association was found between the severity of PV and the highest quartile of DDS, even after adjusting for the potential confounders. Another reason for the lack of significant association between the highest categories of DDS and the odds of disease severity may be related to having higher anthropometric indicators, such as weight, waist circumference, high systolic blood pressure, and smoking in individuals of this category of DDS. In this regard, it has been reported that dietary diversity per se is directly related to obesity if it leads to increased intake [44]. Several studies have reported a significant positive association between DDS and energy intake [45–48]. In these studies, the DDS was estimated based on the Food Guide Pyramid, which is not a tool for controlling energy intake. These reports are also consistent with our results. On the other hand, it is also known that obesity is associated with oxidative stress and low-grade systemic inflammation, which may be associated with a delay in vasculogenesis, granulation, and re-epithelialization of wound sites [49]. One meta-analysis reported that the severity of psoriasis, as a localized autoimmune skin disease, has been associated with the severity of obesity [50]. In other words, the co-existence of pemphigus vulgaris and obesity as an inflammatory state should be given more attention, and other effective strategies are needed to control the progression of complications in this group of patients. In particular, in PV patients with severe disease, chronic corticosteroid intake leads to appetite increase and obesity. Such trends have also been seen in our results. Therefore, it seems that having higher anthropometric factors, blood pressure, and smoking in patients present in this category, impairment of skin and mucosal lesion recovery leads to tissue necrosis, which can influence patients’ dietary habits and dietary choices. Also, it is possible that the inflammatory status of patients in the fourth quartile masks the effects of dietary diversity.

In this study, we observed that the PDAI score was higher in patients who had a higher dietary diversity score in dairy products than in individuals who had less diversity in this food group. Among the food items in the dairy group, low-fat products of milk, yogurt, and cheese were consumed more by the participants in our study (data not shown). Therefore, this relationship cannot be attributed to the adverse effects of fat in dairy products. Among the mentioned products, yogurt was consumed more frequently than the other two groups. Yogurt, a popular fermented milk product in Iran, contains histamine and other biogenic amines, which have been hypothesized to play a role in inflammatory responses [51]. Previous studies have suggested that histamine metabolism and enzyme activity (Histamine-N-Methyltransferase, Diamine Oxidase) might influence inflammation and skin lesions under autoimmune conditions [52–55]. However, our study did not measure these biochemical markers; such mechanisms have been proposed in the literature and warrant further investigation. Therefore, both the variety and specific types of foods may be relevant to disease severity. Our study includes some limitations. First, the cross-sectional design of the study does not allow a cause-and-effect conclusion. Second, there is a possibility of misclassification, recall bias, and over- or under-reporting of dietary intake in this study due to the use of FFQ. Third, there is a possibility of selection bias due to the diagnosis and selection of PV patients by a dermatologist. Fourth, not considering the dietary fat intake in the calculation of the DDS, therefore, their effects have been eliminated. Fifth, we lacked data on the economic situation, which could affect access to diverse food. Sixth, failure to account for seasonal variation. Despite these limitations, this is the first study to examine the relationship between DDS and the severity of disease in PV patients in the Middle East. In addition, this association was also investigated in terms of patients’ oral lesion status. Also, the use of logistic regression models and potential confounding factors was simultaneously controlled.

## Conclusion

This study suggests that moderate dietary diversity may reduce the severity of pemphigus vulgaris in Iranian patients. Promoting balanced dietary diversity, focusing on nutrient-rich foods, could serve as a supportive strategy for managing PV severity. Patients are encouraged to adopt varied diets under nutritional guidance to optimize health outcomes. Further prospective studies are needed to confirm these findings.

## Supplementary Information

Supplementary Material 1.

## Data Availability

All of the data are available with a reasonable request from the corresponding author.
