# Supplementary material for: Association of dietary diversity score and severity of pemphigus vulgaris: a cross-sectional study
Source: BMC Nutr. 2025 Nov 11;11:210. doi: 10.1186/s40795-025-01193-0 (PMC12606824; doi:10.1186/s40795-025-01193-0)
Supplement: Supplementary file 1 — Supplementary Material 1. [file 40795_2025_1193_MOESM1_ESM.docx]

**168-Item Food Frequency Questionnaire (FFQ)**

| **Code** | **Food Item** | **Serving Size** | **Measurement Unit** |
| --- | --- | --- | --- |
| 1 | Lavash bread (traditional/machine-made) | 1 palm-sized piece | piece |
| 2 | Barbari bread | 1 palm-sized piece | piece |
| 3 | Sangak bread | 1 palm-sized piece | piece |
| 4 | Taftoon bread | 1 palm-sized piece | piece |
| 5 | Packaged/Fancy breads | 1 slice | slice |
| 6 | Whole wheat bread | 1 slice | slice |
| 7 | Other breads (Koddi, Roghani, etc.) | 1 small piece | piece |
| 8 | Cooked rice | 3 tablespoons | tbsp |
| 9 | Cooked pasta | 3 tablespoons | tbsp |
| 10 | Cooked noodles | 3 tablespoons | tbsp |
| 11 | Wheat flour | 1 cup | cup |
| 12 | Cooked barley | 3 tablespoons | tbsp |
| 13 | Cooked lentils | 3 tablespoons | tbsp |
| 14 | Cooked bulgur | 3 tablespoons | tbsp |
| 15 | White beans | 3 tablespoons | tbsp |
| 16 | Chickpeas | 3 tablespoons | tbsp |
| 17 | Fava beans | 3 tablespoons | tbsp |
| 18 | Soybeans | 3 tablespoons | tbsp |
| 19 | Mung beans | 3 tablespoons | tbsp |
| 20 | Beef/veal | 1 medium piece (80g) | piece |
| 21 | Lamb meat | 1 medium piece (80g) | piece |
| 22 | Ground meat | 2 tablespoons | tbsp |
| 23 | Chicken with skin | 1 medium piece (100g) | piece |
| 24 | Chicken without skin | 1 medium piece (100g) | piece |
| 25 | Fish (except canned tuna) | 1 medium piece (100g) | piece |
| 26 | Canned tuna | 1 small can (80g) | can |
| 27 | Hamburger | 1 patty (100g) | piece |
| 28 | Sausage | 1 piece (50g) | piece |
| 29 | Liver | 1 medium piece (80g) | piece |
| 30 | Heart and kidney | 1 medium piece (80g) | piece |
| 31 | Tripe | 1 piece (100g) | piece |
| 32 | Low-fat milk (<2.5%) | 1 glass (240ml) | glass |
| 33 | Whole milk (>2.5%) | 1 glass (240ml) | glass |
| 34 | Plain yogurt | 1 small bowl (150g) | bowl |
| 35 | Strained yogurt | 100g | grams |
| 36 | Creamy yogurt | 100g | grams |
| 37 | Feta cheese | 1 matchbox-sized (30g) | piece |
| 38 | Cream cheese | 1 matchbox-sized (30g) | piece |
| 39 | Animal fat | 1 tablespoon | tbsp |
| 40 | Traditional ice cream | 1/2 glass | glass |
| 41 | Industrial ice cream | 1/2 glass | glass |
| 42 | Butter | 1 teaspoon | tsp |
| 43 | Margarine | 1 teaspoon | tsp |
| 44 | Kashk (dried yogurt) | 2 tablespoons | tbsp |
| 45 | Lettuce | 1/2 glass chopped | glass |
| 46 | Tomato | 1 medium | piece |
| 47 | Cucumber | 1 medium | piece |
| 48 | Fresh herbs | 1 handful | handful |
| 49 | Chopped herbs (for kuku) | 1/2 glass | glass |
| 50 | Zucchini | 1 medium | piece |
| 51 | Eggplant (cooked) | 1 medium | piece |
| 52 | Celery (cooked) | 1/2 cup | cup |
| 53 | Potato | 1 medium | piece |
| 54 | French fries | 10 pieces | pieces |
| 55 | Green peas (cooked) | 1/2 cup | cup |
| 56 | Green beans (cooked) | 100g | grams |
| 57 | Raw carrot | 1 medium | piece |
| 58 | Cooked carrot | 1 medium | piece |
| 59 | Garlic | 3 cloves | cloves |
| 60 | Raw onion | 1 medium | piece |
| 61 | Fried onion | 2 tablespoons | tbsp |
| 62 | Cabbage (all types) | 1/2 cup chopped | cup |
| 63 | Bell pepper | 1 medium | piece |
| 64 | Raw spinach | 20 leaves | leaves |
| 65 | Cooked spinach | 1/2 cup | cup |
| 66 | Turnip | 1 medium | piece |
| 67 | Mushrooms (cooked) | 1/2 cup | cup |
| 68 | Green chili pepper | 1 medium | piece |
| 69 | Corn | 1 ear | piece |
| 70 | Tomato paste | 1 tablespoon | tbsp |
| 71 | Pickles (specify type) | 100g | grams |
| 72 | Salted vegetables | 100g | grams |
| 73 | Salted cucumber | 1 medium | piece |
| 74 | Melon | 1/4 medium | piece |
| 75 | Cantaloupe | 1/4 medium | piece |
| 76 | Watermelon | 1 slice (200g) | slice |
| 77 | Apricot | 1 medium | piece |
| 78 | Cherry | 1 medium | piece |
| 79 | Plum | 1 medium | piece |
| 80 | Peach | 1 medium | piece |
| 81 | Nectarine | 1 medium | piece |
| 82 | Green grape | 1 small bunch | bunch |
| 83 | Fresh fig | 1 medium | piece |
| 84 | Dried fig | 1 medium | piece |
| 85 | Grape | 1 small bunch | bunch |
| 86 | Kiwi | 1 medium | piece |
| 87 | Grapefruit | 1 medium | piece |
| 88 | Orange | 1 medium | piece |
| 89 | Persimmon | 1 medium | piece |
| 90 | Mandarin | 1 medium | piece |
| 91 | Pomegranate | 1 medium | piece |
| 92 | Quince | 1 medium | piece |
| 93 | Apple | 1 medium | piece |
| 94 | Sour cherry | 1 medium | piece |
| 95 | Strawberry | 1 medium | piece |
| 96 | Lemon (sweet) | 1 medium | piece |
| 97 | Lemon (sour) | 1 medium | piece |
| 98 | Grapefruit juice | 1 glass | glass |
| 99 | Orange juice | 1 glass | glass |
| 100 | Apple juice | 1 glass | glass |
| 101 | Melon juice | 1 glass | glass |
| 102 | Cranberry | 1/2 cup | cup |
| 103 | Fresh pineapple | 1 slice | slice |
| 104 | Canned pineapple | 1 slice | slice |
| 105 | Raisin | 2 tablespoons | tbsp |
| 106 | Honeydew melon | 1/2 cup diced | cup |
| 107 | Fresh mulberry | 1/2 cup | cup |
| 108 | Dried mulberry | 2 tablespoons | tbsp |
| 109 | Dried peach | 2 slices | slices |
| 110 | Dried apricot | 2 pieces | pieces |
| 111 | Green olive | 5 pieces | pieces |
| 112 | Black olive | 5 pieces | pieces |
| 113 | Solid vegetable oil | 1 teaspoon | tsp |
| 114 | Liquid vegetable oil | 1 teaspoon | tsp |
| 115 | Animal oil | 1 teaspoon | tsp |
| 116 | Mayonnaise | 1 teaspoon | tsp |
| 117 | Peanut | 10 kernels | pieces |
| 118 | Almond | 10 kernels | pieces |
| 119 | Walnut | 1 whole | piece |
| 120 | Pistachio | 10 kernels | pieces |
| 121 | Hazelnut | 10 kernels | pieces |
| 122 | Sunflower seeds | 1 tablespoon | tbsp |
| 123 | Pumpkin seeds | 1 tablespoon | tbsp |
| 124 | Crackers | 2 pieces | pieces |
| 125 | Yazdi cake | 1 piece | piece |
| 126 | Homemade cake | 1 slice | slice |
| 127 | Other cakes | 1 piece | piece |
| 128 | Dry sweets | 1 piece | piece |
| 129 | Wet sweets | 1 piece | piece |
| 130 | Tea | 1 glass | glass |
| 131 | Sugar cube | 1 cube | piece |
| 132 | Sugar | 1 teaspoon | tsp |
| 133 | Rock candy | 1 piece | piece |
| 134 | Honey | 1 teaspoon | tsp |
| 135 | Jam | 1 teaspoon | tsp |
| 136 | Chocolate (specify type) | 1 piece | piece |
| 137 | Candy | 1 piece | piece |
| 138 | Rock sugar | 1 piece | piece |
| 139 | Cheese puff | 1 handful | handful |
| 140 | Potato chips | 1 handful | handful |
| 141 | Gaz | 1 piece | piece |
| 142 | Sohan | 1 piece | piece |
| 143 | Noql | 1 piece | piece |
| 144 | Caramel | 1 teaspoon | tsp |
| 145 | Homemade halva | 1 tablespoon | tbsp |
| 146 | Piroshki | 1 piece | piece |
| 147 | Coffee/Instant coffee | 1 glass | glass |
| 148 | Carbonated drinks | 1 bottle | bottle |
| 149 | Industrial fruit juice | 1 glass | glass |
| 150 | Lemonade | 1 glass | glass |
| 151 | Salt | 1 pinch | pinch |
| 152 | Black pepper | 1 pinch | pinch |
| 153 | Turmeric | 1 pinch | pinch |
| 154 | Cinnamon | 1 pinch | pinch |
| 155 | Saffron | 1 pinch | pinch |
| 156 | Vinegar | 1 teaspoon | tsp |
| 157 | Lemon juice | 1 teaspoon | tsp |
| 158 | Verjuice | 1 teaspoon | tsp |
| 159 | Tomato sauce | 1 tablespoon | tbsp |
| 160 | Pizza sauce | 1 tablespoon | tbsp |
| 161 | Salad dressing | 1 tablespoon | tbsp |
| 162 | Ketchup | 1 tablespoon | tbsp |
| 163 | Mustard | 1 teaspoon | tsp |
| 164 | Pickle juice | 1 tablespoon | tbsp |
| 165 | Doogh (yogurt drink) | 1 glass | glass |
| 166 | Aromatic herbs | 1 pinch | pinch |
| 167 | Food supplements | 1 tablet | piece |
| 168 | Other (specify) | - | - |
